# Should the poultry red mite *Dermanyssus gallinae* be of wider concern for veterinary and medical science?

**DOI:** 10.1186/s13071-015-0768-7

**Published:** 2015-03-25

**Authors:** David R George, Robert D Finn, Kirsty M Graham, Monique F Mul, Veronika Maurer, Claire Valiente Moro, Olivier AE Sparagano

**Affiliations:** Faculty of Health and Life Sciences, Northumbria University, Newcastle upon Tyne, NE1 8ST UK; Technology Centre, Cawood Selby, YO8 3TZ UK; Wageningen UR Livestock Research, Wageningen, The Netherlands; Research Institute of Organic Agriculture FiBL, Frick, Switzerland; Ecologie Microbienne, UMR CNRS 5557, USC INRA 1364, VetAgro Sup, FR41 BioEnvironment and Health, Université Claude Bernard Lyon 1, Villeurbanne, France; Coventry University, Vice-Chancellor Office, Coventry, CV1 5FB UK

**Keywords:** Gamasoidosis, Dermanyssus gallinae, Avian mite dermatitis, Host expansion, Non-host feeding

## Abstract

The poultry red mite *Dermanyssus gallinae* is best known as a threat to the laying-hen industry; adversely affecting production and hen health and welfare throughout the globe, both directly and through its role as a disease vector. Nevertheless, *D. gallinae* is being increasingly implemented in dermatological complaints in non-avian hosts, suggesting that its significance may extend beyond poultry. The main objective of the current work was to review the potential of *D. gallinae* as a wider veterinary and medical threat. Results demonstrated that, as an avian mite, *D. gallinae* is unsurprisingly an occasional pest of pet birds. However, research also supports that these mites will feed from a range of other animals including: cats, dogs, rodents, rabbits, horses and man. We conclude that although reported cases of *D. gallinae* infesting mammals are relatively rare, when coupled with the reported genetic plasticity of this species and evidence of permanent infestations on non-avian hosts, potential for host-expansion may exist. The impact of, and mechanisms and risk factors for such expansion are discussed, and suggestions for further work made. Given the potential severity of any level of host-expansion in *D. gallinae*, we conclude that further research should be urgently conducted to confirm the full extent of the threat posed by *D. gallinae* to (non-avian) veterinary and medical sectors.

## Background

All animals and plants are susceptible to attack by parasites, with most being at least relatively host-specific [1]. Host specificity is by no means universal amongst ectoparasites, however, with some of the most significant species (e.g. mosquitoes and ticks) displaying highly generalist host ranges spanning multiple taxonomic classes [2,3].

In domesticated birds, ectoparasitic mites are a particular issue with *Dermanyssus gallinae* being ubiquitous as a poultry pest throughout much of the globe [4]. Though *D. gallinae* are reported to be avian-specific, albeit infesting more than 30 species of wild birds [5], increasing reports of attacks on non-avian hosts may be indicative of host expansion. Such events are not uncommon among invertebrates, being most often recorded in phytophagous insects. The dipteran *Tephritis conura*, for example, has been recently observed to have expanded its host range in N. Britain to include marsh thistle (*Cirsium palustre*) as well as its ‘standard’ host plant melancholy thistle (*Cirsium heterophyllum*) [6]. A Kenyan population of the *Brassica* ‘specialist’ *Plutella xylostella* provides an even more striking example of inter-family host expansion, having been recorded as infesting peas in 1999, causing heavy losses in this leguminous crop thereafter [7]. For haematophagous insects fewer examples of expansion exist. Nevertheless, numerous studies support generalism in host choice as having evolved from specialism, countering the argument that the latter is a dead-end strategy, and supporting host expansion *per se* as plausible in all specialist feeders [1]. Increased travel and trade, coupled with present and expected impacts of climate change, can be expected to facilitate host-expansion events further in many species, increasing encounter rates with novel hosts and potentially favouring parasite virulence [8]. Increasing densities of humans and associated livestock/companion animals may make medical and veterinary systems particularly susceptible to host expansion events, where increased host occurrence logically favours rising encounter rates with novel parasites [8].

Interestingly, *D. gallinae* has already been found to ‘switch’ more readily between avian hosts of different species than several other related species within the same genus [9]. When removed from hens and offered canaries as a host, *D. gallinae* readily switched between the two, whereas *Dermanyssus longipes* could not. *Dermanyssus carpathicus* was able to switch between hosts, but only after suffering high initial losses not seen with *D. gallinae* [9]. This apparent tendency for higher switching success may reflect the generally broader host range of *D. gallinae* as compared to other species in the genus *Dermanyssus* [5].

Increasing reports of bird-mite attacks on humans and mammalian companion animals (see following Chapters) suggest that avian mite ectoparasitosis/dermatitis (gamasoidosis) may be of increasing medical and veterinary concern. Though several species of bird mite from multiple genera may be responsible for gamasoidosis, *D. gallinae* is most commonly implemented as the causal agent. The aim of this paper was to review past and current cases of *D. gallinae* infestation in non-poultry hosts and, based upon both this information and knowledge of mite biology and ecology, to explore whether *D. gallinae* should be considered as a present or emerging threat to wider veterinary and medical health. Though other avian mites are not explicitly considered, reference to other species is made for comparison.

## Review

### The poultry red mite, *Dermanyssus gallinae*

An in depth review of *D. gallinae* as a poultry pest, including sections on its biology and ecology has recently been published in the Annual Review of Entomology series [4]. Whilst there would be little to merit repeating this information in detail, a brief account of this mites’ life history traits and current accepted significance is still required to place *D. gallinae* in context as a pest *per se*.

**Figure 1:**
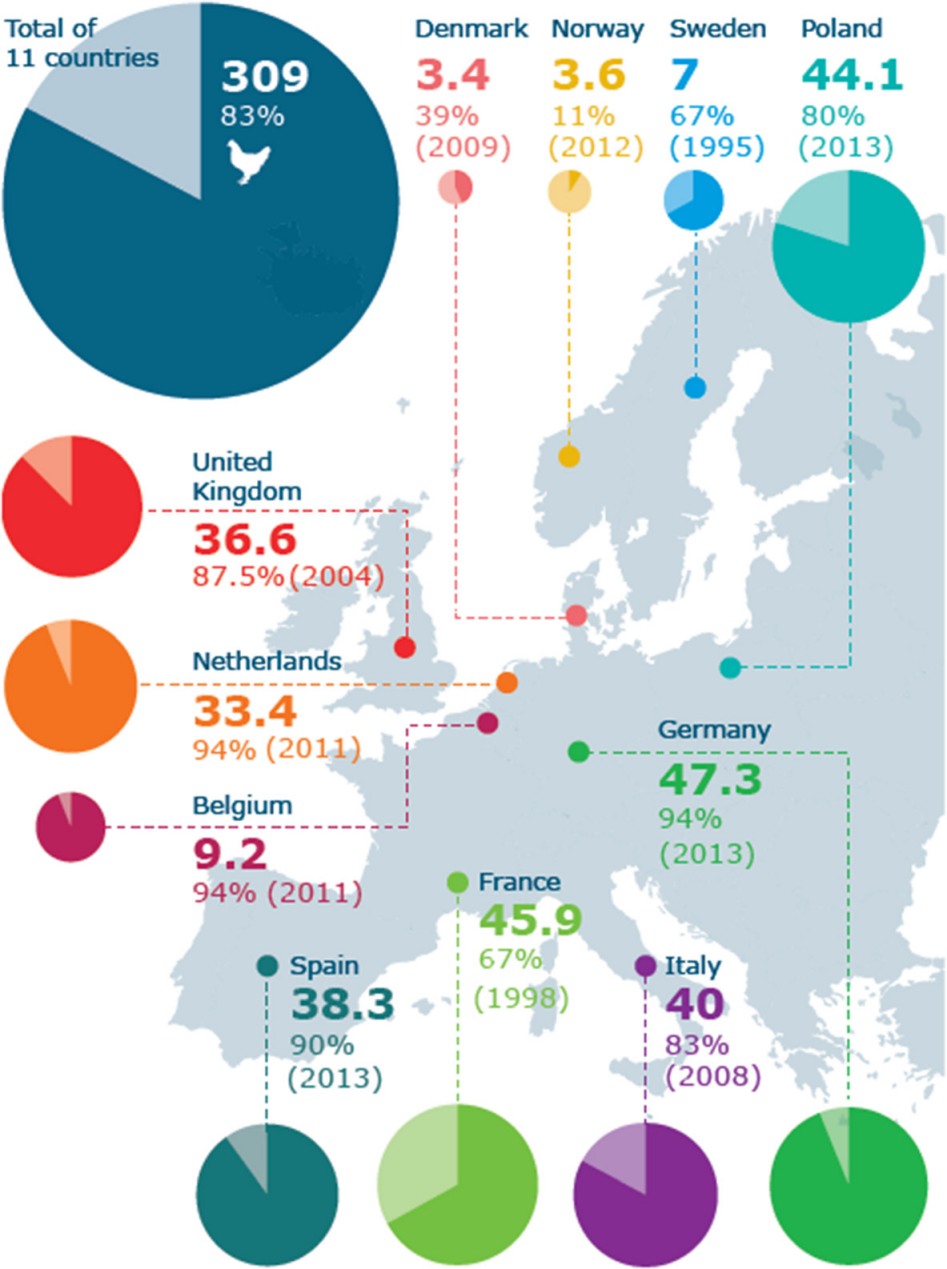
**Number of laying hens per country in millions (2012) and the percentages of farms infested by** ***Dermanyssus gallinae*** **.** Image reproduced from Mul; ©Wageningen UR Livestock Research.

*D. gallinae* poses a significant threat to egg laying hens in many parts of the world, including the US, Europe, Japan and China [10-12]. In Europe infestation rates average more than 80% (see Figure 1), with costs associated with both control and production losses estimated at €130 million per year for the EU egg industry [13]. Production losses are driven by stress to birds and mite populations that may be so high as to result in anaemia and even death of hens by exsanguination [14-16]. Infestation can also lead to declines in egg quality (through increased shell thinning and spotting) and egg production [10,15,17]. Even small mite populations may have significant impact as *D. gallinae* may serve as a disease vector [18-20], with any individual mite potentially harbouring multiple pathogens [20]. Although the absolute vector competence of *D. gallinae* is unconfirmed, their potential to spread disease should not be underestimated [18].

**Figure 2:**
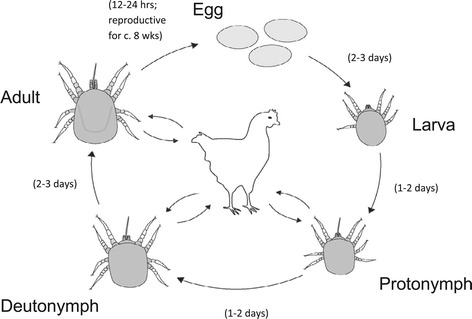
**Life cycle of** ***Dermanyssus gallinae*** **.** Eggs are laid in clutches (4–8 eggs) in refugia where larvae may remain without feeding prior to their first moult. Each female may lay up to eight clutches of eggs in-between feeding bouts, typically laying around 30–50 eggs in a lifetime. Image adapted from Maurer [39].

The rapid life cycle of *D. gallinae* undoubtedly contributes to its status as a pest. Complete development from egg to adult typically occurs over two weeks, though may take place in less than half this time [21,22] (Figure 2). Temperatures of 10-35°C and high relative humidity (>70%) facilitate *D. gallinae* reproduction and development [22,23] and weekly doubling of populations is possible in egg-laying facilities where these conditions are often met [22,24]. Resulting *D. gallinae* densities typically reach 50,000 mites per bird in caged systems, though can escalate to 500,000 mites per bird in severe cases [16].

In egg-laying facilities *D. gallinae* are notoriously difficult to control for multiple reasons, one of these being the tendency of mites to seek refuge in poultry house sub-structures when not feeding. The majority of the *D. gallinae* lifecycle is spent off the host where mites aggregate together in response to both thigmokinesis and pheromone cues [25,26]. From these refugia *D. gallinae* locate their hosts using a combination of temperature stimuli, chemical signals and responses to vibration and carbon dioxide [27-30]. Once upon a host, mites feed for short periods of up to an hour, doing so every 2–4 days and typically (though not exclusively) during periods of darkness [31,32]. Larvae do not feed and though adult males may, they are thought to do so only intermittently [10]. Though feeding is required to permit reproduction and development of some stages, *D. gallinae* may survive for extended periods without a blood meal, permitting survival for up to 9 months when hosts are absent [23]. The development of pesticide resistance in *D. gallinae* also makes control challenging. Resistance to carbamates and pyrethroids has been widely reported and observed in *D. gallinae* from the UK [33,34], Sweden [35], France [36] and Italy [37]. In a survey of British farms published in 2004, more than 60% had experienced acaricide-resistant infestations [38] and figures have likely worsened since [4] (Table 1).

**Table 1:** **Bacterial and viral pathogens ‘associated’ (see table) with** ***Dermanyssus gallinae***

	**Pathogen**	**Association**	**Related references**
Bacteria	Salmonella gallinarum	Isolated from mites	[40]
	Pasteurella multocida	Transmission demonstrated	[41]
	Erysipelthrix rhusiopathiae	Isolated from mites	[42]
	Listeria monocytogenes	Isolated from mites	[43]
	Coxiella burnetii	Transmission demonstrated	[44]
	Nocardia brasiliensis	Isolated from mites	[19]
	Mycoplasma synoviae	Isolated from mites	[20]
Viruses	Newcastle disease	Isolated from mites	[45]
	Fowlpox virus	Transmission demonstrated	[46,47]
	St. Louis encephalitis	Isolated from mites	[48-50]
	Tick bourne encephalitis	Isolated from mites	[51]
	Eastern equine encephalitis	Transmission demonstrated	[52]
	Western equine encephalitis	Transmission demonstrated	[53]
	Venezualan equine encephalitis	Transmission demonstrated	[54]

Table based on information originally published by Valiente Moro et al. [18,55] and updated with data from Chu et al. [20].

## Veterinary significance

As an avian mite recorded from numerous bird hosts it is of little surprise that *D. gallinae* may pose a threat to domestic fowl other than poultry [5]. Companion birds, such as hobby pigeons and budgerigars are also at risk and in canaries *D. gallinae* has even been linked to infection with the bacteria *Chlamydia psittaci* [56].

Though not necessarily commonplace, reports of *D. gallinae* associated with non-avian companion animals do exist. Several references have been made in the literature to suggest that *D. gallinae* will feed from dogs and cats [57-59], with mites also assigned as the causal agent of dermatitis in a 16 year-old domestic horse [60]. *D. gallinae* have also been recovered from goats during skin sampling for mange mites [61], and from mice resident in poultry houses [62]. Such reports, however, do not necessarily confirm infestation of these species; *D. gallinae* may, for example, have been present on goats/mice without feeding from them. Even in work confirming *D. gallinae* as the cause of equine dermatitis, it’s important to note that the horse in question was housed in close proximity to poultry providing the opportunity for this condition to have arisen through repeated adventitious feeding, rather than permanent infestation. Nevertheless, under more controlled laboratory conditions, work supports that *D. gallinae* can and will feed from both mice and rabbits [63], with other work confirming permanent infestation of rodents (gerbils) in the absence of birds that may have otherwise served as a primary host [64]. In later work the ability of *D. gallinae* to subsist on the blood of numerous vertebrate species was demonstrated where these mites “*engorged in vitro on the blood of quail, chickens, sheep, calves, pigs, and rabbits*” [65]. This same work, however, showed that when offered blood of these different animals through different skin membranes “*mites fed satisfactorily only through the skin of birds*.” [65].

The above work perhaps suggests that the skin surface presents more of a barrier to mammalian feeding in *D. gallinae* than non-avian blood. Based on the above reports, however, it seems that this barrier can be overcome, with (at least) adventitious feeding *in vivo* perhaps representing a first step towards host expansion, as occasionally evidenced by permanent infestations on non-avian, seemingly primary hosts.

## Medical significance

According to data presented in Table 2, reports of gamasoidosis have increased in frequency in recent years, particularly in residential settings in association with synanthropic birds. Further reports have appeared in the literature since this data was compiled, with *D. gallinae* recently confirmed as the causal agent of gamasoidosis in five members of a Serbian household [66]. Though numerous cases of gamasoidosis, typically linked to nearby feral birds’ nests and often resulting in dermatological complaints of one kind or another, have been reported for *O. sylviarum* or avian mites in general, *D. gallinae* are most commonly identified as the causal agent (Table 2). *D. gallinae* have also been reported as posing a risk to poultry workers, so much so that this work proposes their presence as an ‘occupational hazard’ [67]. For *D. gallinae* at least, this body of literature, though currently small, confirms ingestion of human blood [68], propensity for persistent infestation when feeding on human blood alone [69] and geographically wide-spread occurrence on a global scale. That *D. gallinae* is assigned responsibility for the majority of gamasoidosis cases is perhaps unsurprising, with laboratory study demonstrating that these mites can be induced to feed upon humans, albeit at low levels, whereas other avian-ectoparasitic mites (*Ornithonyssus* (syn. *Bdellonyssus*) spp) cannot [63] (though see [70]).

Though reports of gamasoidosis are still relatively uncommon, unpublished accounts suggest that in some areas (such as Hawaii) bird mites *per se* have become strongly associated with humans over a relatively short period (<10 years), this being indicative of host expansion (Eco Smart Pest Control, personal communication). More generally, cases of gamasoidosis have been reported since the 17th century [71], documented in the leading medical literature since at least the 1920s [71,72] and reviewed in the last 15 years [64], yet the full extent of gamasoidosis as a threat to human health has still to be explored through empirical research.

The potential medical significance of *D. gallinae* is exacerbated by the fact that these mites can carry and transmit zoonotic diseases of both bacterial and viral origin (Table 1). Though the vector capacity of *D. gallinae* is still an emerging science, mite-bird transmission has been demonstrated in a number of cases [55], increasing the likelihood that relevant diseases carried may also be passed from birds to mammals, humans included. Examples of diseases spread to humans through bird mite vectors are rare in the literature, though transmission of spirochetes, rickettsiae, salmonellae, bartonellae, pasteurellae, sporozoa, hemogregarines, flagellates, and filariae have all been suggested [73]. More recent evidence supports acquisition of *Bartonella* via *Dermanyssus spp* [74]. Worryingly, in a preliminary survey of one internet user group, comprised of past and present gamasoidosis sufferers, more than a third of cases reported associated contraction of Lyme disease, *Bartonella* and/or *Babesia* [75]. Fungal infection was also reported as an associated condition, though it is unclear if this resulted from infestation, or was a pre-existing ‘risk factor’ rendering those affected susceptible to avian mites (see later). Accounts also reported persistent infestations, lasting for many years in extreme cases, despite varied and vigorous treatment interventions. Though hyper-sensitisation could explain symptom persistence, an average infestation longevity of >3 years [75] suggests this to be unsatisfactory as an explanatory hypothesis in all cases.

**Table 2:** **Cases of human attack by avian mite species documented in scientific literature from 1936 to 2013**

**Mite species**	**Details**	**1936-1961**	**1962-1987**	**1988-2013**
Dermanyssus gallinae	Residential	6 [68,76-79]	1 [80]	18 [64,81-89]
	Hospitals	-	6 [90-94]	1 [95]
	Office spaces	-	2 [96,97]	2 [82]
	Occupational*	-	-	4 [69,81,98,99]
Dermanyssus spp. or other species	Residential	-	1 [100]	2 [74,101]
	Occupational	-	-	1 [102]
Ornithonyssus sylviarum/Ornithonyssus spp.	Residential	1 [103]	4 [70,104-106]	8 [64,81,107-111]
	Hospitals	-	1 [94]	-
	Occupational	-	-	1 [112]
Avian mite complex	Residential	3 [73,113,114]	1 [115]	2 [86,116]

Figures show number of independent cases (by mite population), though any given reference may provide multiple cases from a single mite population. *‘Occupational’ includes hobby poultry keepers.

## Discussion

### Scale of the threat

Despite its potential significance, little research had been conducted on the threat of gamasoidosis to non-avian animal and human health, with the bulk of work being formed of case studies documenting occurrence only. Where medical significance is concerned, this is in stark contrast to work undertaken with other (primarily) veterinary ectoparasites of medical concern (e.g. biting flies and ticks); this probably reflects the historically low prevalence of gamasoidosis in comparison.

Diagnosis of gamasoidosis is difficult, whether the mite species involved is *D. gallinae* or otherwise. Considering that at least 25 species of *Dermanyssus* have been described [9], even confirming species within this single genus is troublesome. Several authors have tried to analyse the synanthropic versus wild-environment species, also considering how host-*Dermanyssus* species were organised. Molecular phylogeny studies found that environmental conditions (such as the use of acaricides or pesticides on farms) can influence *D. gallinae* populations, which may consequently show higher diversities regionally than between countries [9,117]. At least two *D. gallinae* clades have been described to date, showing that populations in poultry farms can be organised into several lineages [118]. Work in Sweden and Norway identified several haplotypes of *D. gallinae*, finding wild-type and syanthropic mites to be genetically distinct [119]. This apparent genetic plasticity, coupled with minimal cross-breeding between syanthropic and wild-type mites, may lend itself to host-switching in *D. gallinae*, allowing populations to quickly adapt to novel, even non-avian hosts.

In cases of human infestation, positive identification of species (or at least functionally similar groups based on life-history patterns) and recommendation of suitable treatment requires an understanding of mite taxonomy and ecology that many healthcare professionals and pest control organisations do not currently possess [88]. Diagnosing infestations based on presenting symptoms (as is often undertaken) is inadequate and a suspected cause of large-scale misdiagnosis for similarly-presenting parasitoses such as scabies and pediculosis, general dermatitis or physiological conditions including delusional ectoparasitosis [64,83,95]. Infestation with other mite species, such as *Demodex* mites, may also present similarly [120] as may conditions related to exposure to mite allergens [121]. Confirming infestations based on blood testing is also difficult, with current techniques only being able to ascertain whether the host is responding to mites *per se*. Developments in this area to uncover host markers specifically for *D. gallinae* would be useful, though may be hampered as these mites are thought to adopt a feeding strategy of minimal interference [122]. Diagnosing *D. gallinae* in companion or livestock animals is likely to be equally problematic, with other ectoparasitic mites (such as the mange mite *Sarcoptes scabiei*) being far more common on these hosts and presenting similarly. Available tools to assist clinicians in diagnosing gamasoidosis *per se* do exist (e.g. [123]), though the extent to which such material is consulted is unknown.

It is consequently difficult to predict the current extent of gamasoidosis and increased effort needs to be focused in this area. We speculate that although persistent infestations are likely to be relatively rare, population development of *D. gallinae* on human, livestock and (non-avian) companion animal hosts may be possible if certain conditions are met, these perhaps relating most crucially to host immunosuppressive function and the consequent breakdown of mite feeding deterrence at the skin surface (see below).

### Associated risk factors

The apparent co-occurrence of gamasoidosis and various immunosuppressive disorders [75] indicates that bird mites are more likely to attack and develop persistent populations on human hosts with a weakened immune response. In other ectoparasitic mites a relationship between increased severity of infestation and immunosuppression is better supported. The primary risk factor for crusted (or Norwegian) scabies in humans, for example, is recognised as immunodeficiency [124]. Host defences are commonly cited as a driver for parasite specificity [2], further suggesting that their breakdown could facilitate attack from a broader parasite fauna. Such a relationship between immunosuppression and gamasoidosis, should it exist, could explain apparent anomalies associated with many reports of this condition; such as why relatively few poultry workers report problems with gamasoidosis and why the condition may affect some members, but not others, of the same household. It would also support special consideration of gamasoidosis as a threat in sectors such as hospitals, neonatal units and nursing homes, particularly among those afflicted by, or receiving immunosuppressive treatment for, conditions such as HIV and cancer, or with natural immunodeficiency as a result of pregnancy or neurological/developmental disorders. Accepting such a relationship also raises the interesting question as to whether apparent increased incidence of gamasoidosis in recent years could be a result of improvements in healthcare allowing for prolonged survival of those suffering from immunosuppression.

According to work presented earlier, the skin surface of mammals appears to represent the limiting factor to *D. gallinae* non-host feeding, with ingestion of mammalian blood through an avian skin membrane being acceptable to these mites, at least in terms of development, moulting and oviposition [65]. Accepting the above link between gamasoidosis and immunodeficiency, it is therefore logical to surmise that a decrease in immune function at the skin surface is sufficient to promote persistent *D. gallinae* infestations on non-avian hosts. Interestingly, many of the respondents to a recent survey [75] reported fungal skin infections co-occurring with *D. gallinae* infestation, which would support a hypothesis that persistent mammalian infestation by this species is only limited by the immune response at the skin surface (with fungal skin infection being indicative of this failing). Among healthy humans antimicrobial agents are produced at the skin surface. These include human β-defensins, cathelicidin LL-37, lysozyme, RNase 7, elafin, psoriasin, dermicin, adrenomedullin, secretory leukocyte protease inhibitor and neutrophil gelatinase-associated lipocalin, that protect the skin by targeting foreign biota [125]. Similar skin-surface products act against mosquitos in a number of ways, repelling, deterring or even confusing (e.g. blocking) host seeking processes [126]. Any breakdown/imbalance in the production of these, or similar products, could be the ‘smoking gun’ for gamasoidosis susceptibility, rendering individuals more acceptable to *D. gallinae* (and, potentially, other avian mites) based on changes in skin surface chemistry. It also deserves note, however, that gamasoidosis can occur in seemingly healthy individuals, affecting entire households equally [66]. This suggests that though immunosuppression may increase susceptibility, it is not necessarily a pre-requisite for infestation. This a deserving and interesting area for future research into this little-studied condition, both in humans and other non-avian hosts.

**Figure 3:**
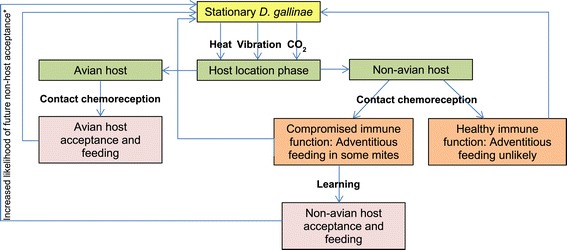
**Theoretical host location, selection and acceptance processes for** ***Dermanyssus gallinae*** **on avian and non-avian hosts.** Yellow: Dormant phase; Green: Host location phase; Orange: Host selection phase; Red: Host acceptance phase. Note the positive feedback loop for non-host acceptance and feeding which if sustained could potentially lead to a heightened chance of accepting non-avian hosts with healthy immune function.

Even in the presence of an immunocompromised novel host, *D. gallinae* would still need to accept a foreign skin surface through which to feed, with preference alone thought to restrain host range in some parasites [2]. In work to develop synthetic skin surfaces for *D. gallinae in vitro* culturing, engorgement through ‘foreign’ membranes is supported [127], though higher feeding rates are typically achieved when these are impregnated with kairomones from an avian host (such as skin or feather extracts) [28,127]. More generalist cues, including temperature, vibration and CO_2_, may also play a role in the host selection process [27,29,30], potentially promoting (at least) attraction to any warm, respiring host. Adaption to novel cues that precede an otherwise appropriate stimulus in the host location/selection process may occur rapidly in invertebrates through ‘learning’ [128], suggesting that *D. gallinae* could ‘learn’ to associate non-host skin with a blood-meal if the host selection process permitted feeding. Thus, we postulate that even low-level exploratory feeding through non-deterrent foreign membranes, such as human skin in immunocompromised subjects, could promote host expansion in *D. gallinae* (see Figure 3), particularly when combined with a seemingly generalist approach to host location (above), and an ability to process a non-avian blood-meal *per se* [63-65,69].

### Treating infestations

Based on the available literature, terminating the majority of *D. gallinae* infestations in humans appears relatively straight-forward once a positive diagnosis has been made. *D. gallinae* are principally regarded as an environmental pest, typically associated with synanthropic birds as their primary hosts, particularly feral pigeons [84]. In most reported cases removal of these birds from nesting or roosting sites in the vicinity of afflicted patients, with or without subsequent acaricide treatment of the area, is sufficient to arrest infestations, with any continued development of infestations on a diet of human blood assumed to be self-limiting. Nevertheless, and as previously noted, *D. gallinae* may develop on human blood [63] and cases of persistent infestation on human hosts do exist [69,75].

Recommended treatments for persistant human infestations with *D. gallinae* (and other avian mites) principally include topical and premise-based pyrethroids, premise-based insect growth regulators and diatomaceous earths, and oral ivermectin, all of which have been reported to fail [75]. Recommending topical treatments for *D. gallinae* (that reside off-host) is inappropriate and unlikely to effectively target and eliminate infestation. The opposite would be true for *O. sylviarum*, however, highlighting the importance of positive diagnosis that extends beyond “gamasoidosis” *per se* in effective treatment prescriptions. Furthermore, it is widely known from poultry research that resistance to pyrethroids, as well as other standard acaricides, is now commonplace in *D. gallinae*, and the effect of diatomaceous earths on this mite may be highly variable [4]. Thus, alternative treatment by novel or bio-pesticides may be more successful in targeting *D. gallinae* [129,130], and perhaps better accepted in a domestic setting for use by either humans or companion animals.

## Conclusion

Though confirmed reports of persistent gamasoidosis in the absence of avian hosts remain rare, those that do exist highlight host expansion potential. Due to a paucity of studies on the topic, the risk of this occurring on a large scale remains unknown. It can be concluded, however, that *D. gallinae* pose a particular host expansion threat due to their genetic plasticity, relatively catholic host location process, willingness to at least feed adventitiously through foreign membranes and ability to process a non-avian blood-meal. That *D. gallinae* often persist in close proximity to man, livestock and/or companion animals is also of concern, optimising opportunistic non-avian feeding events and thus potentially increasing the likelihood of non-avian host acceptance. The continuing rise in global human populations (as well as those of associated livestock and companion animals) can be expected to exacerbate the issue, with resulting increased contact between parasites and novel hosts expected to facilitate host expansion and/or switching events [2].

With the advent of the internet and various user-groups/forums it can be at least tentatively surmised that as a medical condition gamasoidosis is under-represented in the scientific literature [75], this being a probable result of the difficulty in diagnosing this condition. To effectively diagnose and treat gamasoidosis more research is needed. Work is most urgently required to confirm prevalence, determining the mite species involved and potential links to human disease. Also important are investigation of effective treatment interventions, particularly in light of reported issues with *D. gallinae* acaricide resistance in poultry [4] and repeated failure of prescribed treatments for gamasoidosis [75].

## Abbreviation

EU: European Union
